# Identification of alternative splicing events by RNA sequencing in early growth tomato fruits

**DOI:** 10.1186/s12864-015-2128-6

**Published:** 2015-11-16

**Authors:** Yuan Sun, Han Xiao

**Affiliations:** National Key Laboratory of Plant Molecular Genetics, Institute of Plant Physiology and Ecology, Shanghai Institutes for Biological Sciences, Chinese Academy of Sciences, Shanghai, 200032 China

**Keywords:** Alternative splicing, New transcription regions (NTRs), RNA-seq, Transcriptome, Fruit, Tomato

## Abstract

**Background:**

Alternative splicing (AS) regulates multiple biological processes including flowering, circadian and stress response in plant. Although accumulating evidences indicate that AS is developmentally regulated, how AS responds to developmental cues is not well understood. Early fruit growth mainly characterized by active cell division and cell expansion contributes to the formation of fruit morphology and quality traits. Transcriptome profiling has revealed the coordinated complex regulation of gene expression in the process. High throughput RNA sequencing (RNA-seq) technology is advancing the genome-wide analysis of AS events in plant species, but the landscape of AS in early growth fruit is still not available for tomato (*Solanum lycopersicum*), a model plant for fleshy fruit development study.

**Results:**

Using RNA-seq, we surveyed the AS patterns in tomato seedlings, flowers and young developing fruits and found that 59.3 % of expressed multi-exon genes underwent AS in these tissues. The predominant type of AS events is intron retention, followed by alternative splice donor and acceptor, whereas exon skipping has the lowest frequency. Although the frequencies of AS events are similar among seedlings, flowers and early growth fruits, the fruits generated more splice variants per gene. Further comparison of gene expression in early growth fruits at 2, 5 and 10 days post anthesis revealed that 5206 multi-exon genes had at least one splice variants differentially expressed during early fruit development, whereas only 1059 out of them showed differential expression at gene level. We also identified 27 multi-exon genes showing differential splicing during early fruit growth. In addition, the study discovered 2507 new transcription regions (NTRs) unlinked to the annotated chromosomal regions, from where 956 putative protein coding transcripts and 1690 putative long non-coding RNAs were identified.

**Conclusions:**

Our genome-wide analysis of AS events reveals a distinctive AS pattern in early growth tomato fruits. The landscape of AS obtained in this study will facilitate future investigation on transcriptome complexity and AS regulation during early fruit growth in tomato. The newly found NTRs will also be useful for updating the tomato genome annotation.

**Electronic supplementary material:**

The online version of this article (doi:10.1186/s12864-015-2128-6) contains supplementary material, which is available to authorized users.

## Background

Tomato (*S.lycopersicum*) is a model plant for studying fleshy fruit development. Fruit development post anthesis can be divided into four major stages—fruit set, cell division, cell expansion and ripening [1–3]. Early fruit growth in tomato, usually referring to the growth within 2–3 weeks after anthesis, is characterized by rapid cell division and drastic cell expansion. For example, in the small-fruited wild tomato species *S.pimpinellifolium* the cell number of pericarp is doubled within 2 days post anthesis (dpa) and cell division ceases around 5 dpa, whereas cell expansion starting as early as 2 dpa contributes to the remaining fruit growth [2]. Therefore, fruit size largely determined by cell number and cell size is defined predominantly during early fruit growth. In addition, cell expansion during early fruit growth also contributes to the major changes in fruit structure and its biochemical and physiological properties. Several genes regulating the formation of important agricultural traits in tomato have been shown to exert their actions during early fruit growth [4]. For example, *ovate* and *sun*, two major quantitative trait loci (QTLs) controlling fruit shape and the major fruit weight locus *fw2.2* execute their functions mainly during early fruit development [5–7]. The cell wall invertase gene *LIN5* also regulates the formation of solid soluble content during early fruit growth [8]. Several studies of gene expression profiling using microarray have revealed that during tomato fruit set and early growth more than 1000 genes are differentially expressed [2, 9–12], indicating that complex transcriptional regulation, especially of genes related to cell division and hormone biosynthesis and perception, is involved in early fruit growth.

Regulation of gene expression is perceived at multiple levels including transcriptional and post-transcriptional regulation. Alternative splicing (AS) of precursor message RNA (pre-mRNA), which can generate a number of different transcripts from individual multi-exon genes, is one of the important regulatory mechanisms at post-transcriptional level in eukaryotes [13–16]. AS may increase transcriptome complexity and expand genome’s protein coding capacity, having a profound impact on protein functionality, stability and expression levels [15, 17]. By analysis of expressed sequence tags (EST) and RNA-seq data, AS events with various frequencies have been detected in many eukaryotes, including yeast, fungi, plants and animals [18–20]. For example, it has been estimated that more than 95 % of the human multi-exon genes undergo AS [21, 22]. In plants, it has been estimated that 40–63 % of multi-exon genes undergo AS [23–28]. Recently, Chamala et al. have performed a survey on AS patterns in different plant species by computational analysis of EST, mRNA and short reads from public available RNA-seq data, and found that depending on species, 39.1–70.4 % of multi-exon genes produce at least one splice variants in nine plant species including tomato (*S.lycopersicum*), *Medicago truncatula*, soybean (*Glycine max*), common bean (*Phaseolus vulgaris*), rice (*Oryza sativa*), *Arabidopsis thaliana*, poplar (*Populus trichocarpa*), grape (*Vitis vinifera*) and *Amborella* [29].

AS plays important roles in many biological processes, especially in response to environmental stresses [30]. Also, AS is regulated by developmental processes and by external stimuli, such as light [31], temperature [32, 33], salt [34] and pathogens [35]. Wang et al. using RNA-seq assessed the genome-wide changes of AS events during Arabidopsis flower development, and found that AS patterns are similar between different developmental stages [36]. They reported that about 25 % of the expressed multi-exon genes underwent AS and 1716 splice variants were differentially expressed, indicating that AS is regulated by floral development. Developmental regulation of AS events has also been confirmed in maize leaf and poplar xylem [37, 38]. Despite the recent advance in identification of organ-specific AS events and in developmental regulation of AS in few model plants, the AS landscape during fruit development has not yet been obtained from tomato, a model plant for studying fleshy fruit development.

To investigate the AS patterns of early growth fruits, we applied strand-specific RNA-seq to analyze the genome-wide AS events in tomato tissues including seedlings, flowers and early growth fruits at 2, 5 and 10 dpa, and compared the AS patterns in fruits between three characteristic stages of early fruit growth. We found 59.3 % of expressed multi-exon genes underwent AS in at least one of these tissues investigated. Furthermore, the study revealed that there were more splice variants per gene detected in early growth fruits than in seedlings and flowers. We also identified 27 genes showing differential splicing during early fruit growth. In addition, we identified 2507 new transcription regions (NTRs) unlinked to any annotated chromosomal segments, providing a rich resource for future functional genomic analysis in tomato.

## Results

### Identification of AS events in early fruit development

**Fig. 1:**
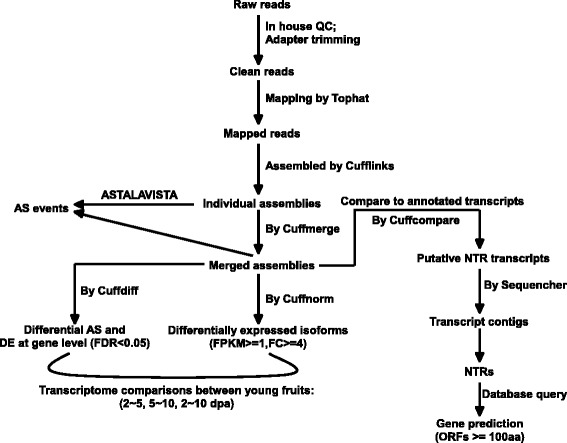
The flow chart of gene expression analysis and AS detection used in this study. Following mapping of the reads to the reference genome (version ITAG2.5), the mapped reads were assembled by Cufflinks. The Cufflinks tools Cuffdiff was used for identification of differentially expressed genes at gene level and differential alternative splicing. Cuffnorm was used for estimating expression levels of individual transcripts assembled by Cufflinks (isoforms). Then a very conservative cutoff (FPKM > =1 and fold change (FC) > =4) was used to select differentially expressed splice variants. AS events were extracted from the merged (fruits) or individual (seedlings and flowers) GTF generated by Cufflinks using the web server tool ASTALAVISTA. For NTR analysis, transcripts assembled by Cufflinks (merged GTF file from all samples) were compared to the annotated ITAG2.4 transcripts and then novel transcripts were analyzed by Sequencher

Early fruit growth in tomato is characterized by active cell division and cell expansion [2]. To investigate the genome-wide AS events in early fruit growth, we performed an RNA-seq analysis on the young developing fruits of *S.pimpinellifolium* LA1589 at 2, 5 and 10 dpa in three biological replicates. In LA1589, the three time points represent three distinctive stages of early fruit development: 2 dpa is the time when fruit set is completed and cell division has concurrently started; at 5 dpa, cell division almost ends; and at 10 dpa, the fruits undergo extensive cell expansion. In addition, RNA-seq was also conducted on seedlings of LA1589 and *S.lycopersicum* cv Heinz1706 as well as anthesis flowers of *S.lycopersicum* LA2397. The three samples were sequenced in one replicate in similar depths with the young fruits (Table 1). The three additional samples were included to access the overall AS patterns in other genotypes and tissues, and the two seedling samples were also used to evaluate the AS detection by mapping of the reads from LA1589 to the reference genome of the cultivated tomato Heinz1706 because there is no annotation yet for the sequenced LA1589 genome. It is thought that reads from LA1589 samples can be readily and has been successfully mapped to the Heinz1706 reference genome because of the small nucleotide divergence between the two genomes [39–43]. Strand-specific transcriptome libraries were constructed and sequenced on a Miseq platform. In total, 43.6 million paired-end reads of high quality in 250 bp length were obtained from the twelve libraries and used for gene expression analysis and AS detection based on the work flow outlined in Fig. 1. The reads were mapped by TopHat2 (version 2.0.12) to the tomato reference genome (version ITAG2.5) [44]. On average, 76.1 % concordant read pairs were uniquely mapped and were kept for further analysis (Table 1).

**Table 1:** Summary of read mapping

Sample	Replicate	Raw read pairs	Mapped read pairs	Percentage
LA4345 seedling		9,733,364	6,724,767	76.9
LA2397 flower		7,122,453	5,189,153	81.9
LA1589				
Seedling		9,464,816	6,228,539	73.6
2 dpa fruit	R1	1,855,723	1,497,635	83.5
	R2	1,477,166	1,191,334	85.0
	R3	1,538,156	1,279,635	86.6
	subtotal	**4,871,045**	**3,968,604**	**81.5 %**
5 dpa fruit	R1	4,061,009	3,248,627	84.9
	R2	3,311,563	2,378,533	78.5
	R3	3,998,480	3,225,436	85.8
	subtotal	**11,371,052**	**8,852,596**	**77.9 %**
10 dpa fruit	R1	2,980,137	2,344,077	81.9
	R2	3,102,963	2,572,168	86.4
	R3	4,456,793	3,576,141	83.2
	subtotal	**10,539,893**	**8,492,386**	**80.6 %**
Total		43,637,807	33,227,506	76.1 %

The numbers in Bold is the sums of the three replicates for 2, 5 and 10 dpa fruit, respectively

**Fig. 2:**
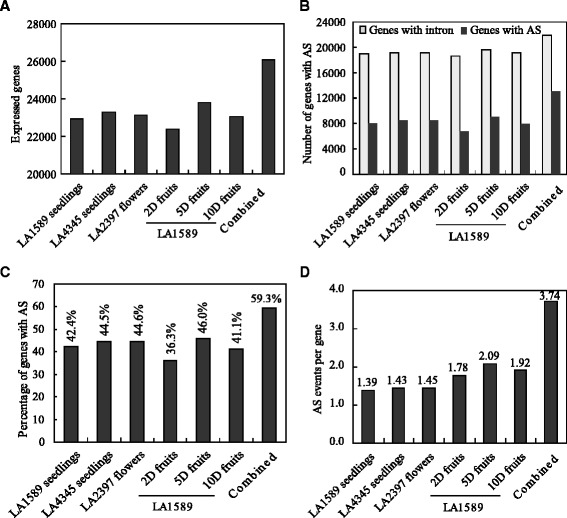
AS events identified in seedlings, flowers and early growth fruits. **a** Numbers of expressed genes in seedlings, flowers and early growth fruits at 2, 5 and 10 dpa. Genes with expression values equal to or higher than 0.1 FPKM in at least one samples were considered expressed. **b** Numbers of expressed multi-exon genes that underwent AS. **c** Frequencies of AS events in seedlings, flowers and early growth fruits. **d** AS events per gene in seedlings, flowers and early growth fruits. 2D fruits represent 2 dpa fruits, 5D fruits for 5 dpa fruits, 10D fruits for 10 dpa fruits

We then used Cufflink program (version 2.2.1) to assemble the aligned reads and to access the transcriptome complexity in early growth fruits. Expression values at gene and isoform levels were estimated based on FPKM (Fragments Per Kilobase of transcript per Million mapped reads). We detected a total of 26,062 genes (75.1 % of the 34,725 annotated genes in ITAG2.4) expressed in at least one of the 12 samples (arbitrary cutoff with FPKM ≥ 0.1). In general, the numbers of expressed genes in seedlings, flowers and fruits range from 22,404 (64.5 %) to 23,821 (68.6 %); the lowest and highest numbers were from 2 and 5 dpa fruits, respectively (Fig. 2a). Based on the current annotation, about 75.5 % (26,216 genes) of the predicted tomato genes have multi-exons. Among them, 21,898 (about 83.5 %) genes were expressed in at least one of these samples. There were similar numbers of multi-exon genes expressed in seedlings, flowers and young fruits at 2, 5 and 10 dpa (Fig. 2b). In the six different tissues, we were able to detect 48,484 splice events from 12,978 multi-exon genes, meaning that 59.3 % of the expressed multi-exon genes were alternatively spliced (Fig. 2c). Overall, the six tissues had 36.3–46 % genes that underwent AS; a slightly fewer AS events were detected in the 2 dpa fruits and the 5 dpa fruits had a relatively higher percentage of genes that underwent AS (Fig. 2c). Although there were similar numbers of genes that underwent AS in seedlings, flowers and young fruits, the averaged numbers of splice variants per gene were higher in fruits than that in seedlings and flowers—the fruits produced 1.78 to 2.09 splice variants per gene, compared to 1.39 and 1.45 in seedlings and flowers, respectively (Fig. 2d). If combining the AS events found in all these samples, there were 3.74 splice variants per gene, indicating that multi-exon genes produce tissue-specific splice variants.

**Fig. 3:**
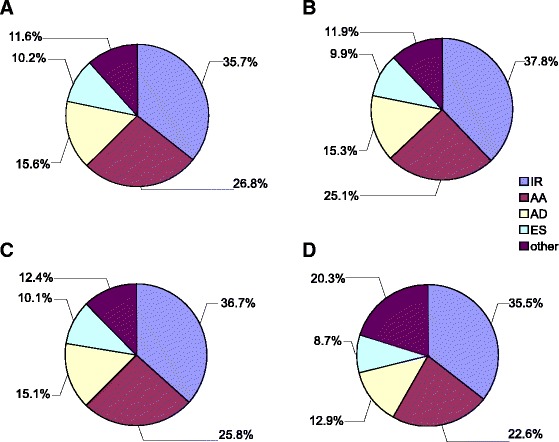
Frequencies of AS types in seedlings, flowers and fruits. **a** Frequencies of different AS types detected in the seedlings of *S.pimpinellifolium* LA1589. **b** Frequencies of different AS types detected in the seedlings of *S.lycopersicum* cv Heinz1706 (LA4345). **c** Frequencies of different AS types detected in the anthesis flowers of *S.lycopersicum* LA2397. **d** Frequencies of different AS types detected in LA1589 fruits. Type information of AS events was extracted from ASTALAVISTA outputs. AS events in the fruits at 2, 5 and 10 dpa were combined. IR, intron retention; AA, alternative acceptor; AD, alternative donor; ES, exon skipping; Others, AS events with more than one of the four basic types

Splice variants are mainly generated by intron retention (IR), alternative splice donor (AD), alternative splice acceptor (AA), exon skipping (ES) and by a combination of the abovementioned four mechanisms. To further investigate the AS patterns of seedlings, flowers and fruits, we used the online tool ASTALAVISTA to extract the AS events from the GTF files generated by the Cufflinks program (version 2.2.1) [45]. The AS events in young fruits at 2, 5 and 10 dpa were combined using the merged GTF file from the mapped fruit-derived reads. Among the AS events identified, IR is the most abundant type (35.5–37.8 %), followed by AA (22.6–26.8 %), AD (12.9–15.6 %) and ES (8.7–10.2 %) (Fig. 3). There are also considerable AS events (classed as others, 11.6–20.3 %) containing more than one of the abovementioned four AS types. Overall, the seedlings of LA1589 and Heinz1706 showed similar AS patterns with LA2397 flowers, whereas the fruits had a higher frequency of mixed AS types (others) and lower percentages of AD and ES types, implicating increased complexity of AS events during early fruit growth. The similar percentages of genes that underwent AS and distribution of AS types between LA1589 and Heinz1706 seedlings also indicate the AS detection by mapping of the reads from LA1589 fruits to Heinz1706 genome is feasible.

**Fig. 4:**
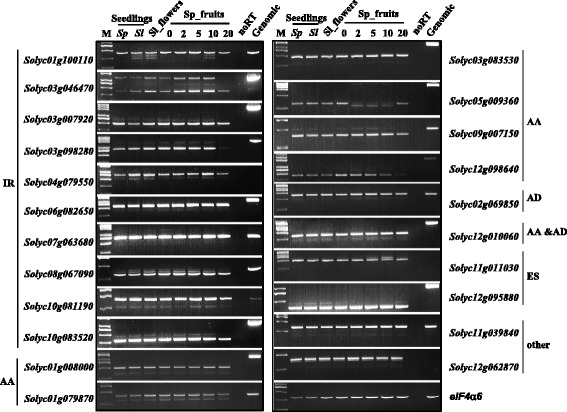
Validation of AS events in different tissues by RT-PCR. Twenty-two multi-exon genes that underwent AS were validated by RT-PCR. RT-PCR analysis was performed on multiple tissues including those used for RNA-seq. *eIF4α6* was used as control. Sl, *S.lycopersicum* cv Heinz1706; Sp, *S.pimpinellifolium* LA1589. noRT, negative control using reaction mixture without reverse transcriptase added as templates. Genomic, genomic DNA of LA1589 as PCR templates. M, DNA marker 2 K Plus II (Transgen, Beijing). IR, intron retention; AA, alternative acceptor; AD, alternative donor; ES, exon skipping; Others, AS events with more than one of the four basic types. Function description and primer information for the 22 genes can be found in Additional file 8: Table S5

To validate the AS events detected by RNA-seq, we performed a reverse transcription-PCR (RT-PCR) analysis on 22 multi-exon genes producing the five types of AS events using RNA samples isolated from seedlings, flowers and fruits at different developmental stages. We were able to detect the expression of these splice variants in multiple samples, and further found that several splice variants showed tissue-specific expression patterns (Fig. 4). For example, both of *Solyc03g046470* and *Solyc05g009360* had one splice variant only expressed in the fruits at 2, 5 and 10 dpa, but not in seedlings and flowers (Fig. 4). Moreover, the RT-PCR analysis showed that the AS events detected in the LA1589 samples were also found in seedlings or flowers of cultivated tomato, except those only expressed in fruit tissues.

### Genes showing differential splicing during early fruit development

Further using the Cufflinks tool Cuffdiff, we identified 27 differential splicing genes which their AS patterns were significantly changed during early fruit development. The 27 genes can be clustered into three different sets based on the expression patterns of their major mRNA isoforms. Set A contains ten genes showing differential splicing between 2 and 5 dpa, eight genes in Set B showed different splicing behaviors between 5 and 10 dpa fruits, and Set C has nine genes displaying differential splicing only between 2 and 10 dpa (Table 2). Most of the 27 genes encode proteins that are likely involved in transcriptional regulation of protein-coding genes and maintenance of protein stability. For example, *Solyc01g008370* and *Solyc05g056310*, encoding a 26S proteasome regulatory subunit and the T-complex protein 1 subunit gamma, respectively, are likely required for protein degradation and protein folding. In addition, *Solyc03g094030*, encoding the mediator of RNA polymerase II transcription subunit 31, also showed differential splicing. Interestingly, a *UPF3* like gene (*Solyc10g044450*) displayed differential splicing between 2 and 10 dpa fruits. UPFs are thought to be important regulators of nonsense-mediated decay (NMD) of premature termination codon-containing (PTC) transcripts generated by AS. This indicates that alternative splicing of some NMD pathway components is regulated by early fruit development.

**Table 2:** Gene showing differential splicing during early fruit development

Gene	Locus	sqrt(JS)	q_value	Description
Set A. Differential splicing between 2 and 5 DPA				
XLOC_019368	SL2.50ch06:41258292-41261163	0.8153	0.0196	
Solyc01g008370.2	SL2.50ch01:2477505-2483463	0.4339	0.0196	26S proteasome regulatory subunit; Mov34/MPN/PAD-1
Solyc02g065080.2	SL2.50ch02:36247331-36257903	0.5335	0.0478	bisphosphoglycerate-dependent phosphoglycerate mutase; Phosphoglycerate mutase
Solyc02g078550.2	SL2.50ch02:43208052-43218654	0.6032	0.0196	RNA polymerase II C-terminal domain phosphatase-like 1; NLI interacting factor
Solyc05g056310.2	SL2.50ch05:65645696-65652967	0.7211	0.0196	T-complex protein 1 subunit gamma
Solyc07g040980.2	SL2.50ch07:51437477-51452251	0.3527	0.0478	Genomic DNA chromosome 5 P1 clone MWD9
Solyc07g052290.1	SL2.50ch07:60781101-60783578	0.5739	0.0196	Membrane-associated zinc metalloprotease family protein expressed; putative membrane-associated zinc metallopeptidase
Solyc09g007390.2	SL2.50ch09:978653-981575	0.6351	0.0196	Mitochondrial import inner membrane translocase subunit TIM14; Heat shock protein DnaJ, N-terminal
Solyc11g008580.1	SL2.50ch11:2764516-2775461	0.6815	0.0196	Ariadne-like ubiquitin ligase; Zinc finger, C6HC-type
Solyc11g066370.1	SL2.50ch11:52124928-52138411	0.5251	0.0196	DNA ligase; ATP-dependent DNA ligase
Set B. Differential splicing between 5 and 10 DPA				
Solyc03g094030.2	SL2.50ch03:55750063-55753767	0.6323	0.0196	Mediator of RNA polymerase II transcription subunit 31; Mediator complex subunit Med31
Solyc04g015190.2	SL2.50ch04:5371123-5375218	0.8097	0.0196	Glucan endo-1 3-beta-glucosidase 5; Glycoside hydrolase subgroup, catalytic core
Solyc06g071770.2	SL2.50ch06:44200874-44205161	0.4640	0.0196	ZZ type zinc finger domain-containing protein (Fragment); Octicosapeptide/Phox/Bem1p
Solyc09g072650.2	SL2.50ch09:65265342-65271839	0.7751	0.0196	Calmodulin-binding protein MPCBP; Tetratricopeptide-like helical; similar to NO POLLEN GERMINATION RELATED 2, NPGR2 of Arabidopsis
Solyc09g082540.2	SL2.50ch09:68259583-68265738	0.4562	0.0478	Tetratricopeptide repeat protein 28; Tetratricopeptide-like helical
Solyc10g005880.2	SL2.50ch10:673775-683075	0.6752	0.0196	Uridine kinase
Solyc10g074750.1	SL2.50ch10:58404701-58410651	0.8325	0.0196	Unknown Protein
Solyc12g088050.1	SL2.50ch12:63541646-63546252	0.6013	0.0478	Rhamnogalacturonate lyase
Set C. Differential splicing between 2 and 10 DPA				
Solyc01g005030.2	SL2.50ch01:44370-54,566	0.7673	0.0196	Serine/threonine-protein kinase 36; SlMAPKKK1
Solyc01g099300.2	SL2.50ch01:89576746-89599770	0.5204	0.0196	MORC family CW-type zinc finger 3; ATP-binding region, ATPase-like; Histidine kinase, DNA gyrase B-, and HSP90-like ATPase family protein
Solyc01g100110.2	SL2.50ch01:90197631-90201738	0.4835	0.0196	Integrin-linked kinase-associated serine/threonine phosphatase 2C; Protein phosphatase 2C
Solyc01g108200.2	SL2.50ch01:95550586-95559361	0.6657	0.0196	similar to AtCYO1, SCO2 | protein disulfide isomerases
Solyc08g077690.2	SL2.50ch08:61596389-61602289	0.8136	0.0196	Chromodomain helicase DNA binding protein 5; SNF2-related
Solyc10g008110.2	SL2.50ch10:2239307-2246349	0.6772	0.0196	Acyl-CoA oxidase 6; Acyl-CoA oxidase
Solyc10g044450.1	SL2.50ch10:26412570-26436844	0.6054	0.0196	Upf3 regulator of nonsense transcripts-like protein B; Regulator of nonsense-mediated decay UPF3
Solyc11g071540.1	SL2.50ch11:54969330-54978414	0.4903	0.0478	Unknown Protein
Solyc11g071700.1	SL2.50ch11:55099644-55120910	0.5709	0.0478	Ubiquitin carboxyl-terminal hydrolase family protein expressed; ubiquitin carboxyl-terminal hydrolase 2

**Fig. 5:**
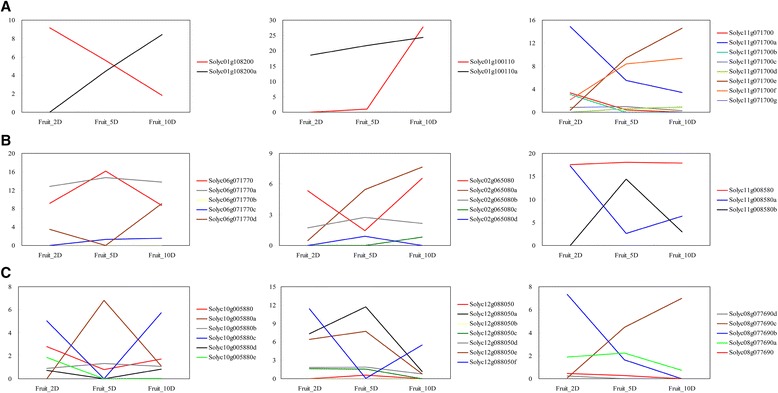
Expression values of mRNA isoforms of differential splicing genes during early fruit growth. **a** Three representative genes showing differential splicing between 2 and 5 dpa. **b** Three representative genes showing differential splicing between 5 and 10 dpa. **c** Three representative genes showing differential splicing between 2 and 10 dpa. Expression values of individual isoforms in FPKM were estimated by Cuffnorm. Description of the nine representative genes can be found in Table 2

We then checked the expression patterns of the individual mRNA isoforms of the 27 differential splicing genes in early developing fruits. Although the 27 genes produced 1–10 alternatively spliced mRNA isoforms, they all had only 2–3 major isoforms expressed at relatively high levels in early growth fruits (Fig. 5 and Additional file 1: Figure S1). For example, the *UPF3* like gene *Solyc10g044450* produced eleven isoforms, but only two isoforms were expressed predominantly during early fruit growth and they had opposite expression patterns. In many cases, the annotated transcripts were expressed either constantly or at low level in early developing fruits. In contrast, their splice variants were differentially expressed during early fruit growth.

### Differentially expressed splice variants during early fruit growth

At gene level, there were 1945 genes differentially expressed (adjusted p-value smaller than 0.05) during early fruit growth, of which 396 and 1737 genes showed differential expression between two consecutive stages of early fruit development, respectively (Additional file 2: Table S1). But as shown in Fig. 5 and Additional file 1: Figure S1, some splice variants showed different expression patterns from its annotated transcript. We then investigated the expression patterns of individual isoforms in 2, 5 and 10 dpa fruits. In these young developing fruits, a total of 29,395 splice variants were detected. Expression level below 1 FPKM is thought to be beyond the limit of protein detection [46–48]. By applying the very conservative cutoff of expression values at 1 FPKM, we found a total of 12,804 splice variants were expressed in these tissues. Among them, there were 6198 and 6564 splice variants from a total of 5206 annotated multi-exon genes showing expression changes by four folds or higher between two consecutive stages at 2, 5 and 10 dpa; these splice variants were considered to be differentially expressed during early fruit growth (Additional file 3: Table S2). When applying the same criteria to the annotated transcripts, only 1059 out of the 5206 genes were differentially expressed, indicating that gene expression during early fruit growth is regulated at multiple layers and expression of splice variants is highly modulated (Additional file 4: Table S3).

**Fig. 6:**
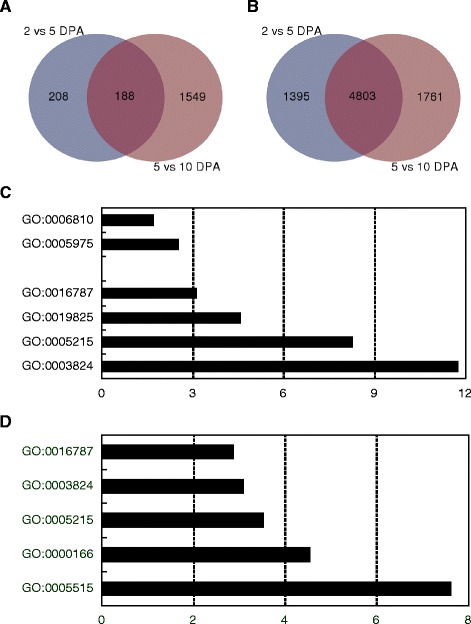
Overrepresented GO terms of differentially expressed genes in early growth fruits. **a** A venn diagram representing the numbers of differentially expressed genes during early fruit growth. **b** A venn diagram representing the numbers of differentially expressed splice variants during early fruit growth. **c** Enriched GO terms for differentially expressed genes during early fruit growth. **d** Enriched GO terms for genes with differentially expressed splice variants during early fruit growth. GO:0005515, protein binding; GO:0000166, nucleotide binding; GO:0005215, transporter activity; GO:0003824, catalytic activity; GO:0016787, hydrolase activity; GO:0019825, oxygen binding; GO:0005975, carbohydrate metabolic process; GO:0006810, transport

Interestingly, we found that at gene level the majority of differentially expressed genes during early fruit growth showed significant changes in expression values from 5 to 10 dpa, but only a small portion of them were also differentially expressed between 2 and 5 dpa (Fig. 6a). In contrast, more than two-thirds of the differentially expressed splice variants had significantly altered expression levels at the three stages (Fig. 6b). The results further demonstrated that AS functions as an important layer of gene expression regulation during early fruit growth.

Several fruit size and shape genes have been shown to exert their functions during early fruit growth. For example, the *SUN* (*Solyc10g079240*) gene encoding an IQD protein family member defines fruit shape early during fruit development and is expressed at higher levels concomitantly [7]. Three isoforms of *SUN* mRNA were detected in the young fruits and the longest splice variant was downregulated after 2 dpa, in contrast to the constant expression of the annotated *SUN* transcript. The shorter *SUN* splice variant was resulted from altered transcription start site at the third intron, encoding a smaller protein, but expressed at low level (FPKM <1.0).

We further conducted an enrichment analysis of GO ontology on the two data sets containing the differentially expressed transcripts at gene and isoform levels, respectively. The analysis revealed that three functional categories—transporter activity (GO:0005,215), catalytic activity (GO:0003824) and hydrolase activity (GO:0016787)—were overrepresented in both of the two data sets (Fig. 6c and d). However, GO term of oxygen binding (GO:0019825) was only enriched in the data set showing differential expression at gene level, whereas two categories of protein binding (GO:0005515) and nucleotide binding (GO:0000166) were overrepresented in that with differentially expressed splice variants. At gene level, genes involved in two biological processes of carbohydrate metabolic process (GO:0005975) and transport (GO:0006810) were overrepresented, but there were no enriched GO terms of biological processes for these genes with differentially expressed splice variants.

### Novel transcription regions

**Fig. 7:**
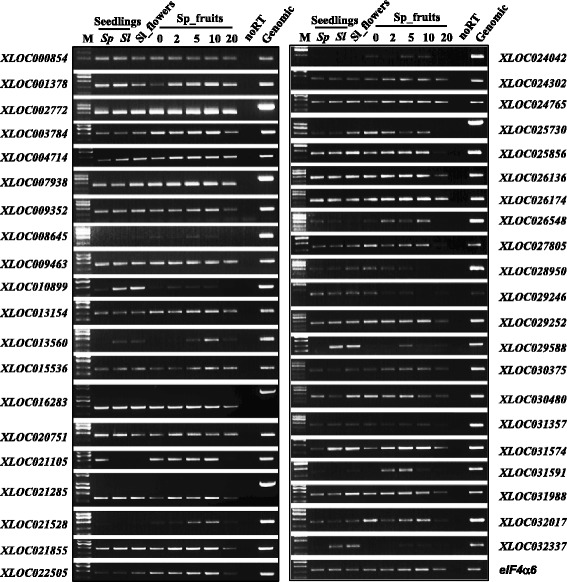
RT-PCR validation of forty-one NTRs. Forty-one NTR transcripts were randomly selected for RT-PCR validation. Sl, *S.lycopersicum* cv Heinz1706; Sp, *S.pimpinellifolium* LA1589. noRT, negative control using reaction mixture without reverse transcriptase added as templates. Genomic, genomic DNA of LA1589 as PCR templates. M, DNA marker 2 K Plus II (Transgen, Beijing). Information of the 41 NTRs can be found in Additional file 4: Table S4 and primer sequences used for RT-PCR were listed in Additional file 8: Table S5

By comparison of all the assembled transcripts identified in our RNA-seq samples to the annotated tomato genes (ITAG2.4), we found a total of 36,397 transcription regions and 3418 of them were chromosomal regions not annotated previously. After assembling all transcripts from the 3418 transcription regions, a total of 2650 unique contigs with lengths from 79 to 13,643 bp from 2507 different chromosomal regions were assembled; these chromosomal regions were considered as new transcription regions (NTRs). More than half of these unique transcript contigs contain multiple sequences of either splice variants or antisense transcripts. We then validated 41 NTRs by RT-PCR using total RNA isolated from different tissues. Transcription was detected for all the 41 NTR transcripts, and we further found that some transcripts showed differential expression during early fruit development (Fig. 7).

In addition, three quarters of the 2650 transcript contigs have nucleotides longer than 500 bp, indicating many of them are likely protein coding sequences. Indeed, blast research against NCBI database revealed that there were 819 NTR transcripts sharing high similarity (e value <1.0e-10) with protein coding sequences from various plant species. In addition, 4 and 474 transcript contigs share high sequence similarity with microRNA precursors and putative long non-coding RNAs (lncRNAs) annotated in NCBI database, respectively (Additional file 5: Table S4). The remaining 1353 NTR transcript contigs share no significant similarity with protein encoding sequences, but 137 of them contain open reading frames longer than 100 amino acids. Therefore, they are likely new protein coding genes (Additional file 6: “Novel protein sequences”). Thus, in addition to the 1690 putative lncRNAs, there are likely 956 new protein coding genes in the tomato genome that have not been predicted.

## Discussion

Recently developed high throughput sequencing technology has allowed effective genome-wide detection of AS events in several plant species. In this study, we used strand-specific RNA-seq to conduct a genome-wide analysis of AS events in cultivated tomato and its direct ancestor *S.pimpinellifolium* and compared the AS patterns between three characteristic stages of early growth fruits. We found that nearly 60 % of tomato multi-exon genes were alternatively spliced, a much higher frequency than the recently estimated 39.1 % [29]. But the frequency is close to the highest ones discovered up to now in Arabidopsis and soybean [24, 25]. More AS events discovered in our analysis is likely benefited from the longer reads we used because longer reads apparently retain more sequence information of the splice junctions. It is also known that the AS frequencies discovered within the same plant species vary considerably among different experiments because of different tissue types and growth conditions as well as different prediction algorithms used. For example, in Arabidopsis, the frequencies of multi-exon genes that underwent AS range from 25 to 61 % [25, 36]. Since AS is affected by tissue types, developmental stages and growth conditions, we would expect higher AS frequencies to be detected in tomato when different tissues at various developmental stages and under different growth conditions are analyzed.

In tomato, early fruit growth contributes to the formation of fruit traits such as fruit morphology and sugar contents [2, 5, 8]. Fruit growth beginning with successful fertilization of ovules has a short duration of cell division followed by a long concomitant cell expansion phase [2]. Transcriptomic profiling has identified a number of differentially expressed genes during fruit set and early fruit growth [2, 9–12]. The identification of 1945 genes differentially expressed in *S.pimpinellifolium* during very early fruit growth (from 2 to 10 dpa) further provides informative knowledge on gene regulation associated with early fruit growth, especially on cell division and cell expansion, which has not been targeted previously. Our gene profiling analysis also revealed that there were a much higher number of genes differentially expressed between 5 and 10 dpa. The result is in agreement with the previous report that from 2 to 5 dpa, *S.pimpinellifolium* fruits mainly undergo cell division, whereas developmental program is shifted to cell expansion in 10 dpa fruits [2]. The expression patterns of genes involved in auxin, gibberellin and cytokinin pathways as well cell cycle regulation are also consistent with the cell division and differentiation in the fruits at the three time points.

Furthermore, our analysis discovered a distinctive AS pattern in early fruit growth, which more AS events per gene were identified in the young fruits compared to other tissues, although the frequencies of AS events in the fruits were close to those in seedlings and flowers. Compared to the number of differentially expressed genes at gene level, more genes showed differential expression at isoform level. We found that 5206 genes had at least one splice variants differentially expressed during early fruit growth, whereas only 1945 of them were differentially expressed at gene level. This indicates that AS plays an indispensible role in regulation of gene expression during early fruit growth. It is worthy to notice that although more genes were differentially expressed during the transition from cell division to cell expansion (5–10 dpa) at gene level, there were similar numbers of splice variants differentially expressed between cell division phase (2–5 dpa) and the cell division-expansion transition. We also identified 27 genes showing significant changes in AS patterns during early fruit growth. It has been shown that the abundance and activity of splicing factors affect the AS profiles of target genes [17]. Among the 27 differential splicing genes, some are likely involved in regulation of transcription and protein stability, such as *Solyc01g008370*, which encodes a 26S proteasome regulatory subunit. The *UPF3* homolog *Solyc10g044450* regulating nonsense-mediated decay of PTC transcripts also showed differential alternative splicing in early fruit growth. This implicates that alternative splicing of some genes involved in maintaining transcript and protein stability is regulated by early fruit development.

High throughput RNA-seq, which can produce millions of reads in a single experiment, has the potential to detect novel transcripts and provides an additional way to facilitate gene prediction. Using RNA-seq, a large number of NTRs unlinked to the annotated loci have discovered in rice [26, 49, 50], soybean [51], grape [52] and Arabidopsis [36, 53]. In this study, we detected 2650 new unique transcripts from 2507 chromosomal regions where no gene has been predicted previously. Consistent with the reported analysis conducted in grape [52], transcripts from more than half of these NTRs are likely lncRNAs, indicating that these putative lncRNAs may play important roles in regulation of early fruit growth in tomato. Furthermore, the remaining about 1000 protein coding genes not predicted previously will be a valuable resource for genomic and genetic analysis of fruit growth in tomato. Therefore, the newly identified NTRs in this study will also facilitate updating the tomato genome annotation in the future.

## Conclusion

In this study, we detected 59.3 % of the expressed multi-exon genes in the tomato genome that underwent AS and found that IR is the most abundant AS type. Comparison of AS events in different tissues revealed that multi-exon genes produced more splice variants in early growth fruits than did in seedlings and flowers. We also discovered that for many genes, transcription was regulated at isoform level rather than at gene level during early fruit growth. In addition, the identification of the more than two thousands of NTRs in this study provides a rich resource for future genomic and genetic analysis in tomato.

## Methods

### Plant materials

The wild relative of cultivated tomato *S.pimpinellifolium* LA1589 and the two cultivate tomatoes *S.lycopersicum* cv. Heinz1706 (LA4345) and LA2397 were obtained from the Tomato Genetics Resource Center at University of California, USA. Plants were grown in phytotrons at 20–25 °C under a humidity of 70–80 % and with daily illumination (150 mE · m^−2^ · s^−1^) for 16 h. Plants were fertilized weekly with all-purpose fertilizer and watered as needed.

### RNA sequencing, read mapping and transcript assembly

Total RNA was extracted by Trizol reagent (Thermo Fisher Scientific, USA) from 7-days-old seedlings, anthesis flowers, fruits at 2, 5 and 10 dpa based on the methods described previously [2]. Paired-end sequencing libraries were created using TruSeq stranded mRNA kit (RS-122-2101, Illumina Inc. USA) and sequenced on Illumina’s Miseq system using 500-cycles Miseq reagent kit (MS-102-2003, Illumina Inc. USA).

Because the cultivated tomato and its closest wild relative *S.pimpinellifolium* LA1589 have only 0.6 % nucleotide divergence in genome sequences [43], reads from LA1589 can be readily mapped to the reference Heinz1706 (*S.lycopersicum*) genome. The 7-days-old seedlings from LA1589 and Heinz1706 were included to make an overall assessment of the impact on AS detection by mapping of the LA1589 reads to the reference genome because of the comparability between the two samples. Therefore, all the 250 bp paired-end reads obtained in this study were mapped to reference genome (version ITAG2.5) using Tophat program v.2.0.12, guided by its corresponding annotation [54]. Following parameters were used: −-read-mismatches 5—read-gap-length 3—read-edit-dist 5—library-type = fr-firststrand—splice-mismatches 0 and GTF. Mapped reads were then assembled by the Cufflinks program (version 2.2.1) using parameters: −GTF-guide –frag-bias-correct –min-frags-per-transfrag 10 [55]. Then, differentially expressed genes at gene level (adjusted *p* value of 0.05 or less) were identified by comparisons between two consecutive stages of early fruit growth using the Cuffdiff tool with parameters used as follows: −frag-bias-correct –multi-read-correct –min-alignment-count 10 –FDR 0.05. The Cuffdiff program also reported genes showing differential splicing and estimated expression values at gene level. The abundance of individual mRNA isoforms was estimated by Cuffnorm using the same parameters as used in Cuffdiff.

NTRs were determined by comparing the assembled transcripts from all mapped reads to the annotated gene regions using the Cuffcompare program with parameters –r –s. New transcript sequences were then extracted and further assembled by Sequencher software (Gene Code Inc. USA) using gapped alignment. Cuffdiff, Cuffnorm and Cuffcompare are parts of the Cufflinks program.

### Identification of AS events

AS events were predicted by the ASTALAVISTA program on the web server (http://genome.crg.es/astalavista/) [45] using the GTF files generated by Cufflinks. The AS events were classed into five types including the four basic types (AA, AD, ES, IR) and others that contain more than one of the four basic types. The output from ASTALAVISTA can be found in the supplementary materials (Additional file 7: “AS landscape.txt”).

### GO ontology enrichment analysis of differentially expressed splice variants

Singular enrichment analysis (SEA) of GO ontology was conducted on differentially expressed genes at gene or isoform level using the online tools AgriGO (http://bioinfo.cau.edu.cn/agriGO/analysis.php). The SEA analysis was performed with statistical test of hypergeometric and multi-test correction by Bonferroni method. Over-represented functional categories of GO terms were selected for those with false discovery rate (FDR) smaller than 0.05.

### RT-PCR validation of AS events and NTRs

Total RNA was extracted by Trizol reagent (Thermo Fisher Scientific, USA) from anthesis ovaries and 20 dpa fruits in addition to the tissues used for RNA-seq based on the methods described previously [2]. After genomic DNA in these RNA samples was removed by RNase-free DNase according to the manufacturer’s protocol (New England BioLabs, USA), the total RNA (1 μg per sample) was used to synthesize first strand complementary DNA using the First Strand cDNA Synthesis Kit (Thermo Fisher Scientific, USA). Ten percent of the reverse transcription products were subjected to PCR analysis. 30 and 35 cycles with annealing temperature at 55 °C for 45 s were used in RT-PCR validation of NTRs and AS events, respectively. Primer information can be found in Additional file 8: Table S5.

### Availability data and materials

All raw reads obtained in this study have been deposited in the NCBI Short Read Archive under Bioproject PRJNA295119.

## Additional files

Additional file 1: Figure S1. Expression values of mRNA isoforms of differential splicing genes during early fruit growth. Expression values (FPKM) of individual mRNA isoforms of 18 additional differential splicing genes during early fruit growth. Expression of mRNA isoforms from the other nine genes is shown in Fig. 3. (PDF 1300 kb) Additional file 2: Table S1. List of differential expressed genes (at gene level) in early growth fruits. Expression values of the 1945 differentially expressed genes in early growth fruits identified by comparison of gene expression between two consecutive stages. Differentially expressed genes were identified by the Cuffdiff program with a cutoff of FDR <0.05. (XLS 735 kb) Additional file 3: Table S2. List of differentially expressed splice variants in early growth fruits. Expression values (FPKM) of the 7959 differentially expressed splice variants in early growth fruits identified by comparison of gene expression between two consecutive stages. Splice variants with expression values of FPKM > =1 and fold changes (FC) > =4 were considered to be differentially expressed. (XLS 2480 kb) Additional file 4: Table S3. List of differentially expressed genes (at isoform level) in early growth fruits. Expression values (FPKM) of the 1059 differentially expressed annotated transcripts in early growth fruits identified by comparison of gene expression between two consecutive stages. Genes with expression values of FPKM > =1 and fold changes (FC) > =4 were considered to be differentially expressed. (XLS 955 kb) Additional file 5: Table S4. Newly identified transcription regions. Description of data: Information about the 2650 unique transcripts assembled from new transcription regions. (XLS 1496 kb) Additional file 6:
**Novel protein sequences.** Protein sequences of unique NTR transcripts. Deduced protein sequences of the 137 putative protein coding sequences from NTRs that share no homology with any protein sequences in NCBI database. (TXT 20 kb) Additional file 7:
**AS landscape.** The output from ASTALAVISTA analysis. All the AS events identified in this study. The data includes information on AS types and chromosomal coordinates. The file is tab-separated with one AS event per line, each one has following fields: <the exon-intron structure of the AS event > <Chromosome > <TranscriptID1 > <Genomic coordinates (variable splice sites #1, variable splice site #2) > <TranscriptID2>. TranscriptID1 and 2 are the two transcripts involved in a given AS event. For the exon-intron structure of the AS event, exon skipping (ES) is indicated by 1-2^,0, alternative donor (AD) by 1^,2^, alternative acceptor (AA) by 1-,2- and intron retention (IR) by 1^2-,0. More detailed description on the AS code (the exon-intron structure) can be found on the ASTALAVISTA webpage (http://genome.crg.es/astalavista/FAQ.html). (TXT 3580 kb) Additional file 8: Table S5. Primers used in this study. Description of data: information of primers used in this study. (XLS 38 kb)
