# Supplementary figures and images for: Identification of alternative splicing events by RNA sequencing in early growth tomato fruits
Source: BMC Genomics. 2015 Nov 16;16:948. doi: 10.1186/s12864-015-2128-6 (PMC4647595; doi:10.1186/s12864-015-2128-6)

**A**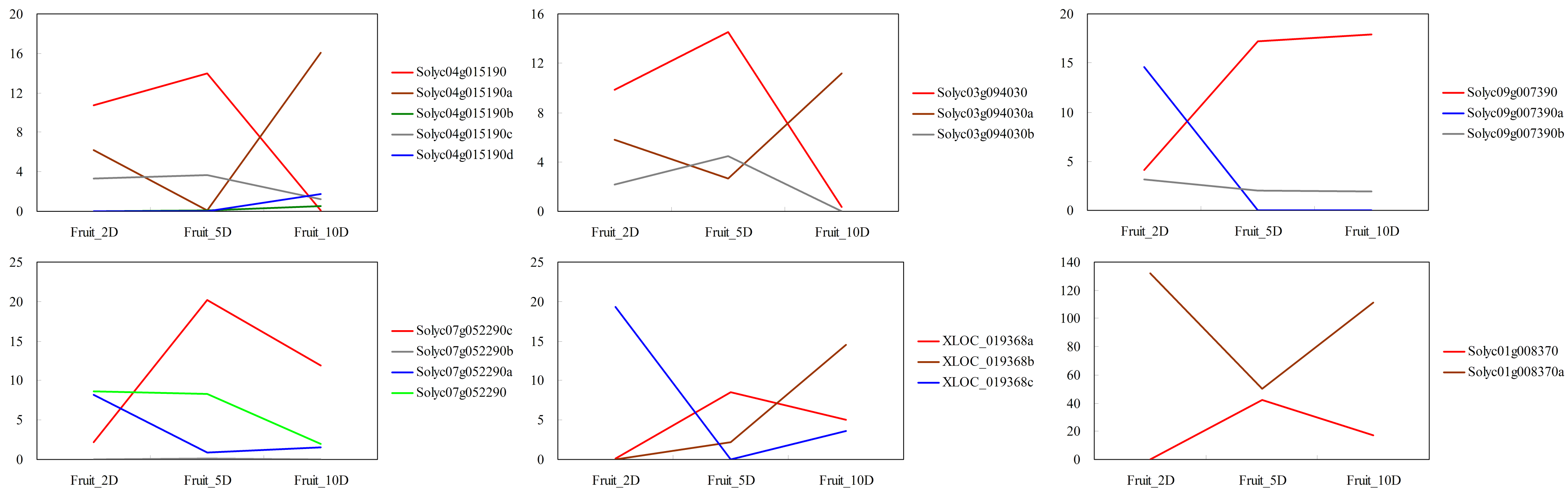**B**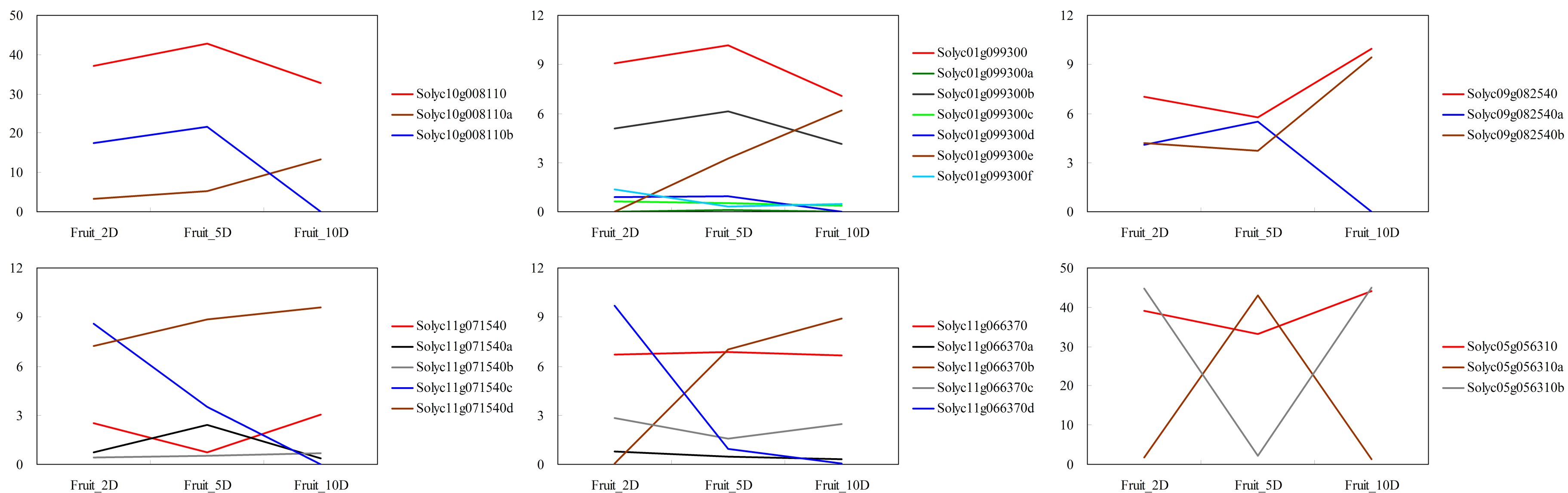**C**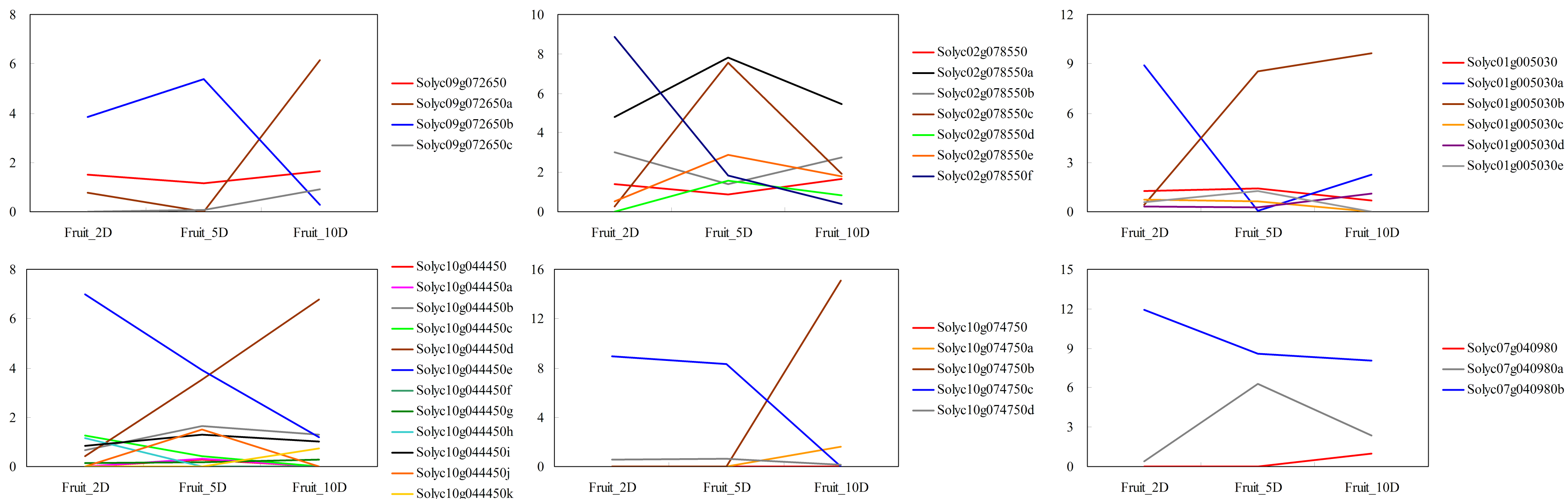

Supplement: Additional file 1: Figure S1. — Expression values of mRNA isoforms of differential splicing genes during early fruit growth. Expression values (FPKM) of individual mRNA isoforms of 18 additional differential splicing genes during early fruit growth. Expression of mRNA isoforms from the other nine genes is shown in Fig. 3. (PDF 1300 kb) [file 12864_2015_2128_MOESM1_ESM.pdf]
